# Supplementary material for: Prioritising non-native fish species for management actions in three Polish rivers using the newly developed tool—dispersal-origin-status-impact scheme
Source: PeerJ. 2024 Oct 31;12:e18300. doi: 10.7717/peerj.18300 (PMC11531750; doi:10.7717/peerj.18300)
Supplement: Supplemental Information 1 [file peerj-12-18300-s001.docx]

**Table S1.** Classification of non-native fish species using the Dispersal-Origin-Status-Impact (DOSI) classification scheme for the Pilica River.

| **Site** | **Species** | **Dispersal Mechanism** | **Origin** | **Status** | **Impact** | **Priority** | **Reference** |
| --- | --- | --- | --- | --- | --- | --- | --- |
| *Pilica River* | *Babka gymotrachelus* | Natural spread, possibly human-mediated  [**D**b*_i,ii_*] | Ponto-Caspian region  [**O**a] | Extending range and abundance  [**S**a*_i,ii_*] | ecological: no effect on native co-occurring species was observed; economic: *NA*; culture: *NA*; health: *NA*  [**I**b*_ii_*] | High Priority | Głowacki et al. 2024. |
|  | *Neogobius fluviatilis* | Natural spread, possibly human-mediated  [**D**b*_i,ii_*] | Ponto-Caspian region  [**O**a] | Extending range and increase abundance  [**S**a*_i,ii_*] | ecological: competitive pressure on native benthic species; economic: *NA*; culture: *NA*; health: *NA*  [**I**a*_ii_*] | Highest Priority | Głowacki et al. 2024. |
|  | *Proterorhinus semilunaris* | Natural spread, possibly human-mediated  [**D**b*_i,ii_*] | Ponto-Caspian region  [**O**a] | Extending range and increase abundance  [**S**a*_i,ii_*] | ecological: no effect on native co-occurring species was observed; economic: *NA*; culture: *NA*; health: *NA*  [**I**b*_ii_*] | High Priority | Głowacki et al. 2024. |
|  | *Percottuss glenii* | Natural spread, ephemeral occurrence in river due to migration from oxbow lakes  [**D**b*_i,ii_*] | East Asia  [**O**a] | Static Range and abundance  [**S**b*_i,ii_*] | ecological: *NA*; economic: *NA*; culture: *NA*;  health: *NA*  [**I**b] | Medium Priority | Głowacki et al. 2024. |
|  | *Ameiurus nebulosus* | Initially introduced in many locations for sport fishing in 19^th^ century, then naturally spread, possibly with human assistance  ephemeral occurrence in river due to migration from ponds and oxbow lakes  [**D**a_i,ii_] | North America  [**O**a] | Deceasing range and abundance  [**S**c*_i,ii_*] | ecological: no effect on native co-occurring species was observed; ecological:*NA*; economic: *NA*; culture:*NA*; health: *NA*  [**I**b*_ii_*] | Low Priority | Głowacki et al. 2024. |
|  | *Carassius gibelio* | Intentional introductions by angling associations and anglers, also escapes from ponds,  then naturally spread  [**D**a_i,ii_] | East Asia  [**O**a] | Extending range and abundance  [**S**a*_i,ii_*] | ecological: competitive advantage over native *Carassius* species; economic: *NA*; culture: *NA*; health: *NA*[**I**a*_ii_*] | Highest Priority | Głowacki et al. 2024. Penczak et al. 2006 |
|  | *Pseudorasbora parva* | Human-mediated, accidentally introduced with stocking material, ephemeral occurrence in river due to escape from fish ponds  [**D**b*_ii_*] | East Asia  [**O**a] | Static range and abundance  [**S**b*_i,ii_*] | ecological: *NA*; economic: *NA*; culture: *NA*; health: *NA*  [**I**b] | Medium Priority | Głowacki et al. 2024. |

**Table S2.** Classification of non-native fish species using the Dispersal-Origin-Status-Impact (DOSI) classification scheme for the Bzura River.

| **Site** | **Species** | **Dispersal Mechanism** | **Origin** | **Status** | **Impact** | **Priority** | **Reference** |
| --- | --- | --- | --- | --- | --- | --- | --- |
| *Bzura River* | *Babka gymotrachelus* | Natural spread, possibly human-mediated  [**D**b*_i,ii_*] | Ponto-Caspian region  [**O**a*_i_*] | Decreasing range and static abundance  [**S**b_i_c_ii_] | ecological: *NA*; economic: *NA*; culture: *NA*; health: *NA*  [**I**b] | Medium Priority | Field observations in 2013, Penczak et al. 2012 |
|  | *Neogobius fluviatilis* | Natural spread, possibly human-mediated  [**D**b*_i,ii_*] | Ponto-Caspian region  [**O**a] | Extending range and increase abundance  [**S**a*_i,ii_*] | ecological: competitive pressure on native benthic species; economic: *NA*; culture: *NA*; health: *NA*  [**I**a*_ii_*] | Highest Priority | Field observations in 2013, Penczak et al. 2012 |
|  | *Proterorhinus semilunaris* | Natural spread, possibly human-mediated  [**D**b*_i,ii_*] | Ponto-Caspian region  [**O**a*_i_*] | Increasing range and static abundance  [**S**b*_i_*a*_ii_*] | ecological: *NA*; economic: *NA*; culture: *NA*; health: *NA*  [**I**b] | High Priority | Field observations in 2013, Penczak et al. 2012 |
|  | *Percottuss glenii* | Natural spread, ephemeral occurrence in river due to migration from oxbow lakes  [**D**b*_i,ii_*] | East Asia  [**O**a] | Static range and abundance  [**S**b*_i,ii_*] | ecological: *NA*; economic: *NA*; culture: *NA*; health: *NA*  [**I**b] | Medium Priority | Field observations in 2013, Penczak et al. 2012 |
|  | *Carassius gibelio* | Intentional introductions by angling associations and anglers, also escapes from ponds,  then naturally spread  [**D**a_i,ii_] | East Asia  [**O**a*_i_*] | Extending range and static abundance  [**S**b_i_a_ii_] | ecological: *NA*; economic: *NA*; culture: *NA*; health: *NA*  [**I**b] | Medium Priority | Field observations in 2013, Penczak et al. 2012 |
|  | *Pseudorasbora parva* | Human-mediated, accidentally introduced with stocking material, ephemeral occurrence in river due to escape from fish ponds  [**D**b*_ii_*] | East Asia  [**O**a*_i_*] | Range and abundance static  [**S**b*_i,ii_*] | ecological: *NA*; economic: *NA*; culture: *NA*; health: *NA*  [**I**b] | Medium Priority | Field observations in 2013, Penczak et al. 2012 |
|  | *Cyprinus carpio* | human-mediated spread, ephemeral occurrence in river due to escape from fish ponds  [**D**a*_i,ii_*] | Danube catchment  [**O**a*_i_*] | Static range and shrinking abundance  [**S**c*_i_*b*_ii_*] | ecological: *NA*; economic: *NA*; culture: *NA*; health: *NA*  [**I**b] | Medium Priority | Penczak et al. 2012  Field observations in 2018 |

**Table S3.** Classification of non-native fish species using the Dispersal-Origin-Status-Impact (DOSI) classification scheme for the Skrwa Prawa River.

| Site | Species | Dispersal Mechanism | Origin | Status | Impact | Priority | Reference |
| --- | --- | --- | --- | --- | --- | --- | --- |
| *Skrwa Prawa River* | *Babka gymotrachelus* | Natural spread, possibly human-mediated  [**D**b*_i,ii_*] | Ponto-Caspian region  [**O**a] | Extending range and increasing abundance  [**S**a*_i,ii_*] | ecological: competitive pressure on native benthic species; economic: *NA*; culture: *NA*; health: *NA*  [**I**a*_ii_*] | Highest Priority | Jażdżewski et al. 2012 |
|  | *Proterorhinus semilunaris* | Natural spread, possibly human-mediated  [**D**b*_i,ii_*] | Ponto-Caspian region  [**O**a] | Extending range and increasing abundance  [**S**a*_i,ii_*] | ecological: no effect on native co-occurring species was observed; economic: *NA*; culture: *NA*; health:*NA*  [**I**b*_ii_*] | High Priority | Jażdżewski et al. 2012 |
|  | *Percottuss glenii* | Natural spread, possibly human-mediated  [**D**b*_i,ii_*] | East Asia  [**O**a] | Extending range and increasing abundance  [**S**a*_i,ii_*] | ecological: *NA*; economic: *NA*; culture: *NA*; health: *NA*  [**I**b] | High Priority | Jażdżewski et al. 2012 |
|  | *Carassius gibelio* | Accidentally introduced with stocking material, then naturally spread, possibly with human assistance  [**D**a_i,ii_] | East Asia  [**O**a] | Extending range and increased abundance  [**S**a_i,ii_] | ecological: *NA*; economic: *NA*; culture: *NA*; health: *NA*  [**I**b] | High Priority | Jażdżewski et al. 2012 |

**References**

Głowacki Ł., Zięba G., Pietraszewski D., Tszydel M., Tybulczuk S., Błońska D., Kruk A., Pyrzanowski K., Leśniak M., Janic B., Penczak T., 2024. Fish fauna in the Pilica river catchment in the sixth decade of study part I. Pilica River. Scientific Annual of the Polish Angling Association. [under review]

Jażdżewski M., Błońska D., Marszał L., Przybylski M., Janic B., Pietraszewski D., Tybulczuk S., Zieliński P., Grabowska J., Zięba G., 2012. Monitoring of fish fauna in the Skrwa Prawa river system: continuation in 2010 and 2011. Scientific Annual of the Polish Angling Association 25, 5–29

Penczak T., Kruk A., Zięba G., Marszał L., Koszaliński H., Tybulczuk S., Galicka W., 2006. Fish fauna in the Pilica river system in the fifth decade of study part I. Pilica River. Scientific Annual of the Polish Angling Association 19, 103–122.

Penczak T., Kruk A., Marszał L., Galicka W., Tybulczuk S., Tszydel M., 2012. Fish fauna regeneration of the Bzura and Ner rivers after industrial sewage reduction. Scientific Annual of the Polish Angling Association 25, 85–93.
