# Supplementary material for: Prioritising non-native fish species for management actions in three Polish rivers using the newly developed tool—dispersal-origin-status-impact scheme
Source: PeerJ. 2024 Oct 31;12:e18300. doi: 10.7717/peerj.18300 (PMC11531750; doi:10.7717/peerj.18300)
Supplement: Supplemental Information 2 [file peerj-12-18300-s002.pdf]

PRIORITY	DEFINITION
**(a) non-native species that spread without human assistance**	
HIGHEST	A population that is expanding and has an impact on the studied ecosystem. These populations are considered the most important to manage due to their active expansion and demonstrated negative effects.
HIGH	A population that is expanding but has currently no observed or only benign impacts. These populations can spread and exert impact elsewhere.
MEDIUM-HIGH	A population that is not expanding but static while having a locally observed impact that warrants monitoring.
MEDIUM	A population that is currently not spreading and has no documented impacts. Due to environmental change, these populations could eventually expand or cause impacts and become problematic and so should be monitored.
**(b) non-native species that spread mainly via human assistance**	
MODERATE-MEDIUM	An impactful population that is expanding due to human activities. These populations rely on human facilitation, and thus, their spead can be hindered by the management of current pathways.
MODERATE	A population that is expanding due to human activities but currently has no observed impacts. These populations rely on human facilitation and, thus, could exert impacts elsewhere. Their spread can be hindered by managing currrent pathways.
LOW-MODERATE	A population with a local impact but is not expanding due to the reliance on human assistance. These populations rely on human facilitation and, thus, could exert impacts elsewhere. Their local impacts warrant monitoring and potential management interventions.
LOW	A population that has no local impact and is not expanding. These populations rely on human facilitation but could develop impacts elsewhere.
